# Efficacy and safety of lenalidomide for refractory cutaneous lupus erythematosus

**DOI:** 10.1186/ar4111

**Published:** 2012-12-07

**Authors:** Josefina Cortés-Hernández, Gabriela Ávila, Miquel Vilardell-Tarrés, Josep Ordi-Ros

**Affiliations:** 1 Medicine Department, Systemic Autoimmune Diseases Unit, Hospital Universitari Vall d'Hebron, Institut de Recerca (VHIR), Universitat Autònoma de Barcelona, Passeig Vall d'Hebron 119-129, 08035 Barcelona, Spain

## Abstract

**Introduction:**

Cutaneous lupus erythematosus (CLE) is a chronic disease characterized by disfigurement and a relapsing course. Thalidomide has proven its efficacy in refractory cutaneous lupus disease, although it is not exempt from significant side effects and frequent relapses after withdrawal. New thalidomide analogues have been developed but lack clinical experience. The aim of this preliminary phase II study was to evaluate the efficacy and safety of lenalidomide in patients with refractory CLE.

**Methods:**

Fifteen patients with refractory cutaneous lupus disease were enrolled in this single-center, open-label, non-comparative pilot trial between January 2009 and December 2010. Oral lenalidomide (5 to 10 mg/day) was administered and tapered according to clinical response. Patients were followed up for a mean of 15 months (range: 7 to 30). Primary efficacy endpoint was the proportion of patients achieving complete response, defined by a Cutaneous Lupus Erythematosus Disease Area and Severity index (CLASI) activity score of 0. Other secondary endpoints included development of side effects, evaluation of cutaneous and systemic flares, and impact on the immunological parameters.

**Results:**

One patient discontinued treatment due to side effects. All remaining patients saw clinical improvement and this was already noticeable after 2 weeks of treatment. Twelve of those patients (86%) achieved complete response but clinical relapse was frequent (75%), usually occurring 2 to 8 weeks after lenalidomide's withdrawal. No influence on systemic disease, immunological parameters or CLASI damage score was observed. Side effects including insomnia, grade 2 neutropenia and gastrointestinal symptoms, were minor (13%). These resolved after withdrawing medication. Neither polyneuropathy nor thrombosis was observed.

**Conclusion:**

Lenalidomide appears to be efficacious and safe in patients with refractory CLE, but clinical relapse is frequent after its withdrawal.

**Trial registration:**

ClinicalTrials.gov: NCT01408199.

## Introduction

Cutaneous involvement in lupus erythematosus disease is common, largely heterogeneous and characterized by a chronic relapsing course [1-3]. As many as 70 to 80% of patients will develop skin lesions at some point during the course of their disease, with a significant proportion being disfiguring and debilitating [1]. Although most patients respond to the standard first-line therapy of topical steroids and antimalarials, approximately 30 to 40% of cases will be refractory to these regimens. For this significant minority, there is no consensus algorithm and a trial and error approach using multiple systemic agents has shown a variable poor response [4-6].

Thalidomide, a glutamic acid derivative, is an agent with a mechanism of action that includes both tumoricidal and immunomodulatory effects. It was first synthesized in 1954 and widely used as a sedative agent, but had to be withdrawn from the market after teratogenic and neurologic effects were established [7]. In recent years, there has been a renewed interest in its use, and currently is also prescribed in various oncologic, dermatologic, and inflammatory conditions, including refractory cutaneous lupus erythematosus (CLE) [8]. In the latter, a significant rapid clinical response has been reported in 80 to 90% of the treated patients [8-17]. However, despite its effectiveness, sustained long-term remission after its withdrawal is low, relapse occurs frequently and it is not exempt from severe side effects that are the main limitation on its continued use [17].

Given thalidomide's vast clinical potential to treat inflammatory and neoplastic conditions, efforts in the mid-1990s led to the development of a promising thalidomide analogue, lenalidomide (CC-5013 or Revlimid™), to improve tolerability and efficacy over the parent drug. In recent years, lenalidomide (5 to 50 mg/day) has been most extensively studied and approved for refractory/relapsing multiple myeloma and myelodysplastic syndromes [18,19].

Thalidomide and its immunomodulatory analogues (IMiDs), first established as agents with antiangiogenic properties, inhibit the production of cytokines (TNF-α), interleukins (IL) 1β, 6, 12 and TNF-α-induced cell surface adhesion molecules (ICAM-1, VCAM-1, E-selectin). In addition, they promote apoptosis, and increase the NK-dependent cytotoxicity by altering natural killer (NK) cell numbers and function [20]. Although both IMiDs have demonstrated similar biological activities, lenalidomide is more potent than thalidomide and therefore achieves responses at lower doses [20-22]. Lenalidomide has also been shown to have a different toxicity profile. Whereas somnolence, constipation and peripheral neuropathy have been less reported [21], myelosuppression occurs more frequently in a dose-independent manner [18,20-22].

Thus, based on its immunomodulatory properties, the favorable toxicity profile and its potential superior efficacy compared to the parent drug, lenalidomide may be an alternative in refractory inflammatory dermatosis such as cutaneous lupus. So far, there is little familiarity with its use in this field and only a small number of patients have been treated with lenalidomide (5 to 10 mg/day) albeit with encouraging results [23,24]. Given the limited clinical experience, its potential beneficial effects and the need to better understand its effect on the systemic facet of the lupus disease, a larger open phase II trial was initiated.

## Materials and methods

### Study design

The purpose of this open-labelled, single centre, phase II pilot study was to establish the efficacy and safety of lenalidomide in refractory CLE. The study protocol was approved by the local research ethics committee of Vall d'Hebron Hospital and was conducted according to ICH good clinical practice and in accordance with the Declaration of Helsinki. All patients gave their written informed consent. The study was registered at the ClinicalTrials.gov (Identifier: NCT01408199).

### Endpoints

The efficacy primary endpoint was the proportion of patients achieving complete response. Clinical response was evaluated by the validated Cutaneous Lupus Erythematosus Disease Area and Severity Index (CLASI) [25]. Response was defined as follows: *complete response *(CR) as a complete resolution of the inflammatory rash (CLASI activity score = 0); *partial response *(PR) by at least a 50% improvement in the CLASI score by week 12 when compared to baseline, and *no response *when no improvement or worsening in the CLASI score was observed at the same time period.

Secondary endpoints included the proportion of patients: developing any adverse event (AE); having a cutaneous relapse, defined as a new CLASI ≥ 4 in those who previously achieved CR following treatment withdrawal; developing a systemic lupus flare, assessed by the Systemic Lupus Erythematosus Disease Activity Index 2000 (SLEDAI) [26] in which a mild flare was defined as a change in SLEDAI score greater than 3 and a severe flare by a change in SLEDAI greater than 12; having an increment in the CLASI damage score [25] compared to baseline or having an increment of anti-dsDNA antibody titres.

An AE was defined as any adverse deviation from the patient's baseline condition during the trial. The events were categorised as mild, moderate or severe. A serious AE (SAE) was defined as an event that was life-threatening, resulted in death, required or prolonged hospitalisation or resulted in persistent or significant disability/incapacity.

Reasons for discontinuing treatment were: common toxicity criteria grade 3 myelosuppression, withdrawal of consent by the patient, pregnancy, life-threatening complications, severe systemic lupus flare requiring alternative treatment or decision by the physician that discontinuation was in the patient's best interest.

### Inclusion criteria

Patients were recruited from a single centre between Jan 2009 and 2010 according to the following eligibility criteria: aged over 18 years; histological proven CLE with or without associated systemic lupus erythematosus (SLE) disease diagnosed according to the American College of Rheumatology (ACR) SLE classification criteria [27]; presence of at least a grade II erythema as assessed by the CLASI activity score [25]; stable prednisone (< 10 mg/day), antimalarial, or immunosuppressive regimens for at least 30 days before the inclusion; refractoriness to at least 3 months of conventional treatment with antimalarials and topical steroids; and finally, at least one of, an involvement of more than 18% of the body surface area calculated according to the 'rule of the nines', a history of severe thalidomide side effects or lack of efficacy following thalidomide therapy. For all patients, there was no known hypersensitivity to thalidomide. Women were excluded from the study if pregnant, lactating or not using adequate contraception. Other key exclusion criteria included active SLE requiring systemic immunosuppressive agents, presence of severe thrombocytopenia (< 30,000/mm^3^), leukopenia (< 2,000/mm^3^) or neutrophil counts below 1,000/μl, known at least 30 days prior to inclusion; previous history of arterial/venous thrombosis; presence of antiphospholipid antibodies; moderate to severe renal impairment (FG < 30 ml/min/1.73 m^2^) and/or progressive renal disease. Psychiatric or social disorder that might interfere with the follow-up or treatment compliance, HIV or viral hepatitis infections were also exclusion criteria. All patients gave written informed consent prior to participation in the study.

### Treatment protocol and assessment

Lenalidomide was started at 5 mg/day for 4 weeks. At that time, if no clinical improvement was observed, using the criteria specified before, dose was increased to 10 mg/day. Otherwise, lenalidomide was sustained at 5 mg/day in case of partial response or decreased progressively monthly until its withdrawal if complete response was achieved. No changes in concomitant baseline medication were allowed.

At entry, patients had a complete medical history review and physical examination, including a detailed neurological assessment. At baseline measurements of full blood counts and immunological parameters (anti-dsDNA, complement, antinuclear antibodies (ANA), anti-extractable nuclear antigen (ENA) and antiphospholipid antibodies) were performed. The evaluation of each patient's rash took into account the initial extension, activity and degree of scarring as assessed by the CLASI score [25]. This score consists of two separate scores: the activity score, which reflects erythema and scaling; and the damage score, which documents scarring and permanent dyspigmentation.

Patients were followed-up monthly for the first 6 months and every three months afterwards, unless clinically required. At each visit, clinical parameters including a detailed neurological examination, the CLASI score, response to therapy, and occurrence of side effects were evaluated. Nerve conduction studies were performed in those patients with previous thalidomide-induced neuropathy or when new neurological symptoms occurred. Laboratory tests (WBC) were performed weekly for the first 2 months and then monthly following drug safety recommendations. Exacerbations of systemic disease during follow-up were evaluated by the SLEDAI score [26].

### Statistical analysis

Statistical analysis was performed using SPSS version 7.5 (SPSS, Inc., Chicago, IL, USA). Descriptive statistics are reported as frequency and percentage for categorical variables and as mean and standard deviation for continuous variables. Categorical and continuous data were compared by means of the *t *test and Mann-Whitney test, as appropriate. The level of statistical significance was set at *P *< 0.05.

## Results

### Baseline patient characteristics

Fifteen consecutive patients with refractory CLE were included. The major subtypes of the recruits were discoid lupus erythematosus (DLE) (60%), lupus profundus (13%) and subacute cutaneous lupus erythematosus (SCLE) (13%). Table 1 shows baseline clinical and serological data. Fourteen of them had been previously treated with thalidomide and either did not respond (n = 4) and/or developed severe side effects (n = 11). Four patients also had a rash covering more than 18% of the body surface area. All patients were female and of Caucasian origin. Age varied between 28 and 53 with a median age of 40 years. Six patients (40%) had an associated systemic disease, of whom five had positive anti-dsDNA antibodies. Photosensitivity was present in seven patients (47%). The mean (± SD) CLASI activity and damage scores at entry were 11 ± 5.9 and 2.33 ± 2.96, respectively. Initial SLEDAI score was 3.26 ± 2.22.

**Table 1:** Patient demographics and clinical characteristics.

Patient number	Sex	Diagnosis	Site involvement	Reason for inclusión	ANA/dsDNA^α^ antibodies	Anti-Ro/SSA/La/SSB antibodies	CLASI A/D	Previous received treatment
1	F	DLE (L)	V-neck, arm	T lack of efficacy, PN	1/640 (123)	+/-	6/2	TCS, OCS, HCQ, T
2	F	Lupus Profundus	Arm, back	T lack of efficacy	1/160 (-)	-/-	7/1	TCS, OCS, HCQ, T
3	F	DLE (L)	Arm	T lack of efficacy	1/640 (-)	-/-	3/1	TCS, OCS, HCQ, T
4	F	ACLE	Face, v-neck	T side effects	1/640 (54)	-/-	6/0	TCS, OCS, HCQ, T, AZA,
5	F	Lupus Profundus	Scalp, face, arms	T side effects	1/640 (144)	-/-	19/6	TCS, OCS, HCQ, MMF, T
6	F	SCLE	Face, v-neck, back	> 18% body surface	1/640 (-)	+/-	19/0	TCS, OCS, MMF
7	F	SCLE	Face, v-neck, hands, arms	T side effects, PN, > 18% body surface	1/640 (83)	-/-	20/1	TCS, OCS, HCQ, AZA, T
8	F	DLE (L)	Face, scalp, ear lobe	T side effects	1/40 (-)	-/-	9/0	TCS, OCS, HCQ, T
9	F	DLE (G)	Face, ear lobe, arms, neck	> 18% body surface, T side effects	1/320 (37)	-/-	13/2	TCS, OCS, HCQ, MMF, T
10	F	LET	Face	T side effects, PN	1/640 (-)	-/-	6/0	TCS, HCQ, T
11	F	DLE (G)	Face, scalp, back	T side effects, > 18% body surface	1/40 (-)	+/-	15/9	TCS, OCS, HCQ, T
12	F	DLE (G)	Face, v-neck, back, scalp	T side effects, PN	1/40 (-)	+/-	17/8	TCS, OCS, HCQ, T
13	F	DLE (L)	Face	T lack of efficacy	1/160 (-)	-/-	3/0	TCS, HCQ, T
14	F	DLE (G)	Face, v-neck	T side effects	1/640 (-)	-/-	11/2	TCS, OCS, HCQ, MMF, T
15	F	DLE (L)	Face, scalp	T side effects	1/40 (-)	-/-	10/3	TCS, OCS, HCQ, T

^α^Normal value of anti-dsDNA antibodies is < 15 IU/ml. A, activity; ACLE, acute cutaneous lupus erythematosus; ANA, antinuclear antibodies; AZA, azathioprine; CLASI, Cutaneous Lupus Erythematosus Disease Area and Severity Index; D, damage; DLE, discoid lupus erythematosus; G, generalized; HCQ, hydroxychloroquine; L, localized; LET, lupus erythematosus tumidus; MMF, mycophenolate mofetil; OCS, oral corticosteroids; PN, polyneuropathy; SCLE, subacute cutaneous lupus erythematosus; T, thalidomide; TCS, topical steroids.

### Clinical efficacy

**Figure 1:**
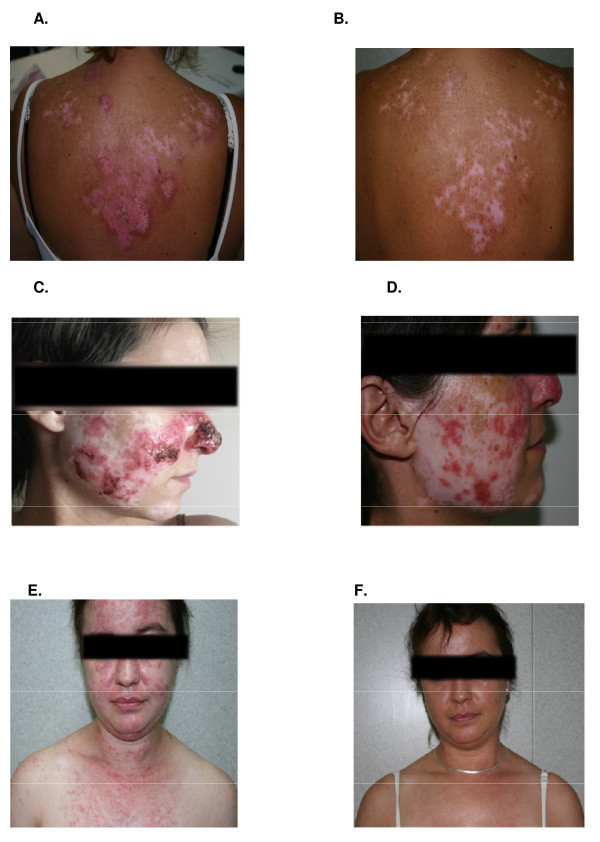
**Clinical responses to lenalidomide therapy**. Discoid lupus erythematosus (patient number 12) on upper back before **(A) **and after 3 months of lenalidomide therapy (5 mg/d) **(B)**. Resolution of the erythema with persistent residual hypopigmentation and some degree of scarring, already existing at baseline from previous cutaneous involvement. Widespread discoid lupus erythematosus (DLE) (patient number 9) involving nose and malar area before **(C) **and after three months of therapy **(D) **resulting in a partial improvement of the erythema and increased hypopigmentation. Widespread subacute cutaneous lupus erythematosus (SCLE) (patient number 6) involving face and V-neck area before **(E)**, and complete resolution after 6 weeks of lenalidomide therapy **(F)**. All patients concerned gave their written consent for the publication of the images.

Clinical response and lenalidomide dosage are shown in Table 2 and Figure 1. One patient discontinued the study after 1 week due to disabling gastrointestinal side effects (patient number 3). Clinical response (combined complete and partial response) was observed in all of the remaining patients (100%) (Figure 1), of whom 12 (86%) achieved a CR. Four of the responders had previously failed thalidomide therapy. A significant clinical improvement was already noticeable at 2 weeks (CLASI activity decreased from 11 ± 5.9 to 4.13 ± 3.66, *P *= 0.0009). Time to CR in those patients with DLE and SCLE was shorter and ranged from 2 to 12 weeks with a median time of 6 weeks. However, in those with lupus profundus, time to CR varied from 4 to 32 weeks with a median time of 13 weeks. At the end of follow-up, two patients (14%) remained in partial response, despite increasing the dose in one case (10 mg/day). Overall, treatment duration ranged from 3 to 30 months with a median of 11 months.

**Table 2:** Summary of clinical response.

Patient number	Clinical outcome	Time to complete response (weeks)	Follow-up period (months)	Maximun dose received (mg)	Relapse	Dose at relapse
1	CR	10	15	5	Yes	5 mg twice weekly
2	CR	4	12	5	Yes	5 mg three times weekly
3	Withdrawn	-	-	5	-	-
4	CR	3	12	5	Yes	5 mg alternate days
5	CR	36	19	5	-	-
6	CR	4	17	5	No	-
7	CR	11	18	5	No	-
8	CR	2	7	5	Yes	5 mg alternate days
9	PR	-	15	10	-	-
10	CR	7	10	5	Yes	5 mg alternate days
11	CR	4	30	5	Yes	5 mg twice weekly
12	CR	4	25	5	Yes	5 mg alternate days
13	PR	-	8	5	-	-
14	CR	12	17	5	Yes	5 mg three times weekly
15	CR	2	10	5	Yes	5 mg alternate days

CR, complete response; PR, partial response.

Patients were followed up for a median of 15 months (range: 7 to 30). Of the twelve patients who achieved complete response, nine (75%) relapsed within 2 to 8 weeks (median 4.4), while reducing medication or after its withdrawal. The lowest dose of lenalidomide at which relapse occurred was 5 mg on alternate days or three times weekly. Five patients had at least two relapses when reducing dose and received two cycles of treatment. All patients responded to the introduction of lenalidomide. Three of the fourteen treated patients (21%) required a long-term maintenance dose. Only two patients, both with SCLE, obtained a sustained remission after withdrawing medication.

**Figure 2:**
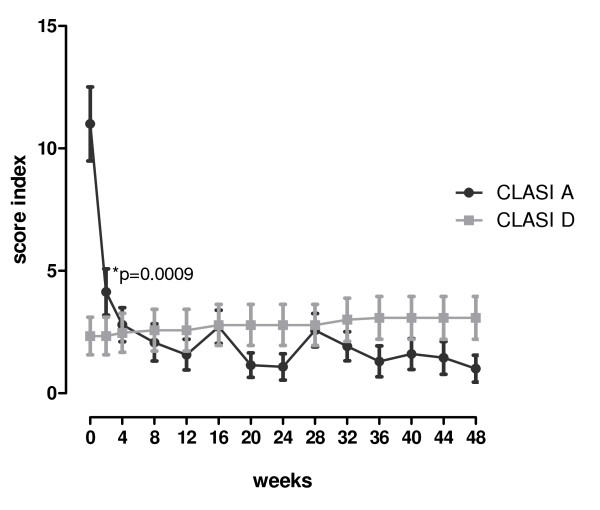
**Cutaneous Lupus Erythematosus Disease Area and Severity Index (CLASI)**. CLASI shows a reduction of the CLASI activity (CLASI A (●)) score with no significant changes in the CLASI damage (CLASI D (■)) score over 12 months of follow-up. A significant improvement of the inflammatory rash was already observed by week 2. Data is expressed as mean ± SEM. *P value refers to the comparison of CLASI scores between two weeks after treatment and baseline.

After one year follow-up, although a slight increment in the CLASI damage score was observed compared with baseline (3.08 ± 3.15 vs. 2.39 ± 2.96, respectively, *P *= 0.398), this was not statistically significant (Figure 2).

### Effect on systemic disease

**Figure 3:**
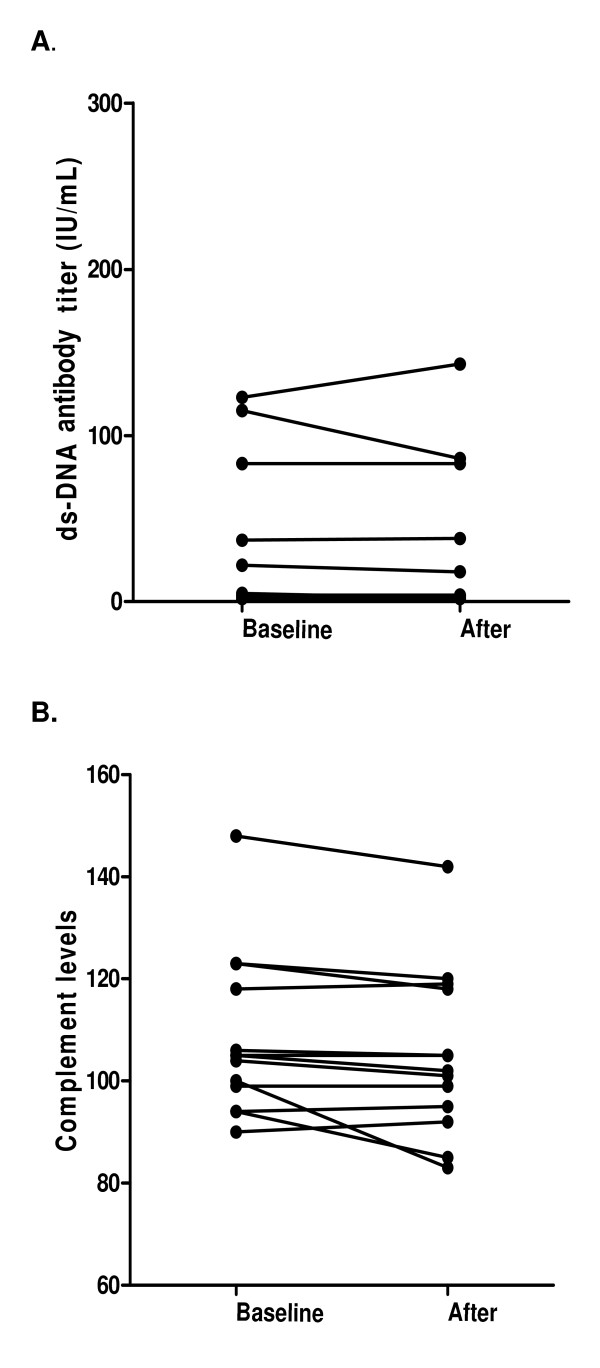
**Effects of Lenalidomide on anti-dsDNA antibody and complement levels**. Comparison of anti-dsDNA antibody titres **(A) **and complement levels **(B) **at baseline and after completing the course of treatment.

Clinically, only one of the seven patients (14%) with associated SLE developed arthralgias and mild arthritis during follow-up (patient number 1). None of the patients with only cutaneous lupus developed systemic symptoms. No significant changes in the mean SLEDAI score were observed during follow-up. Two patients had previous history of lupus nephritis. No new onset or increase in proteinuria was observed. When the impact of lenalidomide was evaluated on the immunological parameters, anti-dsDNA titres did not experience significant changes in patients with or without previous anti-dsDNA antibodies. No effect was observed in complement levels (C3 and C4) either (Figure 3).

### Toxicity

Generally, lenalidomide was well tolerated. Only two patients developed side effects (13%) (Table 3). Patient number 3, one week after medication was initiated, developed severe vomiting, diarrhoea and weight loss that improved after medication was withdrawn and no other factors accounting for this episode were found. Patient number 15 developed insomnia and grade 2 neutropenia (33% neutrophils (1.190/mm^3^)), 2 months into the study while on lenalidomide 5 mg/day. There was no evidence that somnolence was a problem for patients taking the study drug. None of the patients complained of paraesthesia. A repeated electrophysiological study in those patients with previously known thalidomide-induced neuropathy was performed. No worsening was observed. No other evidence of haematological or biochemical toxicities was observed. There were no thrombotic events or ovarian toxicity during the study.

**Table 3:** Adverse events reported during lenalidomide treatment.

Patient	Graded diagnosis	NCI toxicity grade	Causal relationship to lenalidomide treatment
3	Vomiting	Grade 2	Possible
	Diarrhea	Grade 2	Possible
	Weight loss	Grade 2	Possible
	Fatigue	Grade 2	Possible
15	Insomnia	Grade 2	Probable
	Neutropenia		Possible

## Discussion

The purpose of this trial was to investigate the efficacy and safety of lenalidomide, a new thalidomide analogue, in the treatment of refractory CLE. Although in recent years there has been a better understanding of the pathophysiological mechanisms involved in the development of CLE, there has not been a great breakthrough in its treatment. So far, no medication has been approved specifically for this condition and few randomized, placebo-controlled trials, evaluating mainly topical agents in patients with DLE, have been performed [28-30]. Currently, the first-line therapy consists of antimalarials and topical steroids, but for refractory cases there is no consensus, and in most cases, off-label systemic agents licensed for SLE and other immunological diseases are administered.

Thalidomide is one of the few agents with proven efficacy for refractory CLE according to case reports and observational studies [9-17]. However, despite its effectiveness, its use has been limited due to its toxicity profile and the current restricted availability. This trial was encouraged by the partial beneficial effects initially published in two patients with recalcitrant DLE who had been treated with lenalidomide (5 to 10 mg/day) [23] and by our experience using thalidomide in this condition [13,17]. After excluding the patient with disabling side effects, we included a total of 14 patients with refractory CLE. All patients experienced clinical improvement. Complete response was achieved by 86% of them within 2 to 12 weeks after starting lenalidomide. This rate of CR is different from the only report published to date using lenalidomide in CLE. Braunstein *et al. *[24] described a small cohort of five patients treated with the same regimen for 6 weeks. At the end of treatment, in that study, the majority of patients (four out of five) achieved a partial response defined by at least a 4-point decrease in the CLASI score, although most of them experienced a decrease of at least 8 points [24]. Differences in treatment duration, clinical response definition and follow-up between the two studies account for the discrepancies observed. Interestingly, this study also established for the first time preliminary histological and biological factors associated with lenalidomide clinical response. Whereas nonresponders had high CXCL10 cutaneous expression and increased activated circulating CD4+ T cells, responders had reduced glycosaminoglycan and pDCs content with a trend toward increased circulating T regulatory cells. No correlation with the IFN signature was established [24].

As with thalidomide [17], cutaneous relapse was frequent and occurred in 75% of patients within 2 to 8 weeks after tapering dosage or withdrawing medication. Although the study was not designed to establish variations in clinical response between the different histological subtypes of CLE and few patients were included, it was observed that whereas patients with refractory SCLE tended to remain in remission after withdrawing medication, those with DLE or lupus erythematosus tumidus tended to relapse more frequently. Some of the relapsing patients (21%) even required a low long-term maintenance dose (5 mg/alternate days or three times a week) to control the disease.

In the literature to date, a variety of adverse effects have been reported, mainly in patients with haematological conditions and receiving higher doses of lenalidomide. The most commonly described include neutropenia, thrombocytopenia, paraesthesia, skin and gastrointestinal toxicity, and fatigue. Overall, myelosuppression, generally mild, has been the most common, and although is more frequently observed at high doses (25 to 50 mg/day), it can occur in a dose-independent manner [18,22,23,31,32]. In our study, lenalidomide was generally well tolerated and at this dosage displayed a safe profile. The few observed side effects resolved after drug withdrawal. No neurological symptoms were reported and none of the patients with previous thalidomide-induced polyneuropathy experienced worsening in the repeated electrophysiological studies. Athough increased risk of thrombosis has been previously reported [31,32] during lenalidomide treatment, in our study, no thrombotic events were observed. The results need to be interpreted with caution since none of the patients had other associated cardiovascular risk factors, such as smoking or presence of antiphospholipid antibodies.

Still not clear is the effect of lenalidomide on systemic disease. Braunstein *et al. *described an increasing risk of developing a severe systemic lupus flare in their small cohort of patients with one treated patient developing a renal flare. They attributed it to the possible effect of lenalidomide on T cell activation and pDCs [24]. However, no significant clinical or immunological effect of lenalidomide on systemic manifestations of SLE was observed in our larger cohort of treated patients after 15 months of follow-up. Only one patient with known systemic disease developed mild arthritis. None of the patients with CLE progressed to a systemic disease and levels of anti-dsDNA antibodies or complement remained stable during follow-up.

Results were compared with those obtained in our previous cohort of thalidomide-treated patients [17]. Although lenalidomide was better tolerated and had a safer profile, both agents displayed the same efficacy and relapse rates. As lenalidomide is an analogue of thalidomide, the question of resistance to lenalidomide in patients unresponsive to thalidomide arose. Both the literature [21] and our study indicate that clinical benefit from lenalidomide can be obtained in those refractory patients, reflecting mechanistic differences - lenalidomide has greater immunomodulatory properties than thalidomide, whereas thalidomide has greater antiangiogenic activity [20]. Lenalidomide has been found to be more potent in the stimulation of T-cell proliferation and IFN-gamma and IL-2 production than thalidomide, whereas both thalidomide and pomalidomide, another thalidomide analogue, have been found to be more potent at inhibiting sprout formation than lenalidomide when antiangiogenic properties were assessed in a human umbilical explant model (20).

The main limitations of the study are the absence of a randomized group control and the insufficient small sample size to draw conclusions at the histological subtype level since the majority of included patients had DLE. Nevertheless, the present study provides valuable information on the critical issue of treating refractory cutaneous lupus lesions given the significant number of patients studied, the relatively long period of follow-up and the detailed clinical and laboratory information obtained during the follow-up. Further larger controlled trials are required to confirm our observations.

## Conclusions

In conclusion, the present study confirms the efficacy and safety of lenalidomide for refractory cutaneous lupus disease. The benefit of lenalidomide is in reducing disfigurement without thalidomide's toxicity profile.

## Abbreviations

ACLE: acute cutaneous lupus erythematosus; ACR: American College of Rheumatology; AE: adverse event; ANA: antinuclear antibodies; CLASI: Cutaneous Lupus Erythematosus Disease Area and Severity Index; CLE: cutaneous lupus erythematosus; CR: complete response; DLE: discoid lupus erythematosus; ENA: extractable nuclear antigen; LET: lupus erythematosus tumidus; PR: partial response; SAE: serious adverse event; SCLE: subacute cutaneous lupus erythematosus; SLE: systemic lupus erythematosus; SLEDAI: Systemic Lupus Erythematosus Disease Activity Index.

## Competing interests

The authors declare that they have no competing interests.

## Authors' contributions

JCH participated in the design of the study, collected the data, performed data analysis/interpretation and drafted the manuscript. GA participated in data analysis and drafted the manuscript. MVT participated in data interpretation and edited the manuscript. JOR conceived and participated in the design of the study, data interpretation and draft of the manuscript. All authors read and approved the final manuscript

## Authors' information

GA is a rheumatologist at the Lupus Unit at the Department of Autoimmune Diseases, Internal Medicine, University Autónoma of Barcelona, Spain. JCH is a lecturer at the Lupus Unit, Department of Autoimmune Diseases, Internal Medicine, University Autónoma of Barcelona, Spain. MVT is Professor and director at the Department of Internal Medicine, Faculty of Medicine, University Autónoma of Barcelona, Spain. JOR is Professor and director of the Lupus Unit at the Department of Internal Medicine, Faculty of Medicine, University Autónoma of Barcelona, Spain.
